# Dynamics and compositional profiles of human milk oligosaccharides in mothers with gestational diabetes mellitus across lactation

**DOI:** 10.3389/fnut.2026.1816715

**Published:** 2026-06-15

**Authors:** Shihao Guo, Ye Wang, Zhenyu Yang, Di Wang, Mengtong Yang, Xiangnan Ren

**Affiliations:** 1 Chinese Center for Disease Control and Prevention, National Institute for Nutrition and Health, Beijing, China; 2 Key Laboratory of Public Nutrition and Health, National Health Commission of the People’s Republic of China, Beijing, China; 3 Key Laboratory of Human Milk Science, Chinese Center for Disease Control and Prevention, Beijing, China; 4 BYHEALTH Institute of Nutrition and Health, Guangzhou, Guangdong, China

**Keywords:** generalized estimating equations, gestational diabetes mellitus, human milk oligosaccharides, influencing factors, lactation stage

## Abstract

**Purpose:**

This study aimed to systematically clarify the changes in the concentrations of human milk oligosaccharides (HMOs) in mothers with gestational diabetes mellitus (GDM) and the differences from healthy mothers.

**Methods:**

A total of 156 human milk samples were collected from 50 mothers (26 healthy mothers and 24 GDM mothers) in Danyang City at different lactation stages (0–7 days, 8–14 days, 1 month, and 3 months postpartum). By using the method of UPLC–MS/MS to analyse the concentration of HMOs, comparing the changes and the differences of HMO concentrations between healthy mothers and GDM mothers.

**Results:**

Intergroup comparisons of the concentrations of total HMO and 32 individual HMOs were performed at 0–7 days, 8–14 days, 1 month, and 3 months postpartum. After adjusting for covariates (age, BMI, gestational days, mid-trimester blood glucose, and gestational weight gain) by using the generalized estimating equation (GEE), it was found that GDM was significantly correlated with specific HMOs, with the specific results showing that 7 HMOs, including 2′-fucosyllactose (2’-FL), lacto-N-difucohexaose I (LNDFH-I) and 3’-Sialyl-N-acetyllactosamine (3’-SLN), differed significantly in relation to GDM (*P*_all_ < 0.05). Time was closely associated with the total HMOs concentration and other individual HMOs. The total HMOs concentration at 1 month and 3 months postpartum was significantly lower than that in colostrum (*p* < 0.05); Specific HMOs exhibited distinct temporal variation patterns: for example, three kinds of HMOs including 2’-FL showed a downward trend starting from 8–14 days postpartum; the concentrations of eight kinds of HMOs such as lacto-N-fucopentaose II (LNFP-II) and Lacto-N-triose II (LNT-2) decreased significantly at 1 month and 3 months postpartum; HMOs such as 6-sialyllactose (6’-SL) and lacto-N-tetraose (LNT) showed a significant reduction only at 3 months postpartum. Analysis of the interaction between GDM and time indicated that the magnitude of the decrease in the concentrations of 3 HMOs including Disialyllacto-N-tetraose (DSLNT) and N-Acetyllactosamine (LacNAC) in the GDM group at 8–14 days postpartum was significantly greater than that in the healthy mother group.

**Conclusion:**

The total concentration of HMOs in GDM mothers was lower than that in healthy mothers at all time points and showed a downward trend. Meanwhile, the study found that GDM is associated with the concentrations of individual HMOs, and the HMO concentrations in GDM mothers differ from that in healthy mothers at different lactation stages, with all being lower than those in healthy mothers.

## 1 Introduction

Breast milk provides all the nutrients for infants’ growth and development within the first 4–6 months after birth, and this is recognized as the gold standard for infant feeding (1). Human milk oligosaccharides (HMOs) are the third most abundant solid component in breast milk, except the lactose and lipids. They are water-soluble oligosaccharides secreted by maternal mammary glands during lactation (2). HMOs are a class of oligosaccharide compounds composed of 3 to 23 monosaccharide molecules linked by glycosidic bonds. Their definite monosaccharide components include D-glucose (Glc), D-galactose (Gal), N-acetylglucosamine (GlcNAc), L-fucose (Fuc), and sialic acid (Sia) (3), among which N-acetylneuraminic acid (Neu5Ac) is the most predominant form in the sialic acid family (4). There are approximately 200 different structures of HMOs that have been identified in human milk matrix until now (5). HMOs have been shown to reduce infections risk, maintain the balance of intestinal microecology, promote brain and cognitive development, and participate in immune regulation (6, 7). In addition, HMO concentrations are influenced by multiple factors, including genetic background (secretor and Lewis genes), lactation stage, and maternal health conditions such as gestational diabetes mellitus (8–11).

Gestational Diabetes Mellitus (GDM), a common pregnancy-related metabolic disorder, is defined as glucose intolerance first diagnosed during pregnancy, predominantly in the second and third trimesters (12). In China, the prevalence of GDM continues to rise annually, reaching 14.8% (13). GDM exerts multiple adverse impacts on mothers and fetuses. Accumulated evidence demonstrates that GDM alters maternal blood biochemistry and placental morphology (14), which may further affect breast milk composition. Furthermore, it may affect the activities of glycosyltransferases and glycosidases (15), thereby modifying breast milk HMO profiles. However, relevant studies focusing on HMOs alterations in GDM mothers remain scarce, with controversial and inconsistent results. In 2013, Smilowitz et al. (16) collected transitional milk from mothers during the first 2 weeks postpartum and measured oligosaccharide concentrations in the breast milk of GDM mothers and healthy mothers, finding no significant differences between the two groups. In 2021, Ye et al. (17) investigated the impact of GDM on small-molecule metabolites in breast milk at different lactation stages. They found 11 differential metabolites between the GDM group and the healthy mother group in mature milk at 42 days postpartum; compared with healthy mothers, the concentrations of 3-fucosyllactose (3-FL) and lacto-N-difucohexaose II (LNDFH-II) in GDM breast milk were relatively lower, while those of 2’-FL and lacto-N-fucopentaose I (LNFP-I) were relatively higher. At 3 months postpartum, 17 differential metabolites were identified, with lower LNDFH-I in the GDM group. Subsequently, in 2022, Wang et al. (18) analyzed 13 HMOs in colostrum and reported lower sialylated oligosaccharides, particularly 3-sialyllactose (3’-SL), in GDM mothers. In 2023, Zhang et al. (19) confirmed distinct HMO profiles between the two groups throughout lactation. Healthy mothers had higher 2’-FL, LNT, lacto-N-neotetraose (LNnT) and 6’-SL in colostrum, as well as higher Disialyllacto-N-tetraose (DSLNT), LNnT and 3-FL in mature milk. At the beginning of 2023, Dou et al. (20) found higher total HMOs in GDM mothers’ colostrum, with no intergroup differences in transitional and mature milk. Significant disparities in LNnT and 3’-SL (colostrum and transitional milk), and DSLNT (colostrum alone) were noted, with higher levels in the GDM group. In the 2024 study by Zhang et al. (21) demonstrated that healthy mothers had higher total sialylated, non-fucosylated neutral, and fucosylated oligosaccharides in colostrum, whereas DSLNT, 3′-SL and 3-FL were comparable between groups. These studies have focused on the differences in HMOs between GDM mothers and healthy mothers, but the results are inconsistent. This may be attributed to regional variations, gestational comorbidities, heterogeneous GDM phenotypes, detection methods and limitations in sample size. The present study aims to recruit participants in strict accordance with predefined inclusion and exclusion criteria. Compared with previous relevant studies, our study performed longitudinal sampling across multiple lactation stages, adjusted for gestational comorbidities and genetic confounding factors, and adopted generalized estimating equations (GEE) and mixed-effects models for mutual verification, thereby systematically exploring the effects of GDM on 32 types of HMOs. We further integrated our findings with existing literature to summarize and tentatively propose three hypothetical mechanisms underlying GDM’s influence on HMO profiles. In addition, there are relatively few studies on the temporal changes in HMO concentrations in GDM mothers. This study presents the changing trends of individual HMOs in healthy and GDM mothers across different lactation stages, thereby providing more scientific and evidence-based nutritional recommendations and practical guidance for infants of mothers with GDM.

## 2 Subjects and methods

### 2.1 Study subjects

A total of 50 mother-infant pairs from Danyang City, Jiangsu Province, were enrolled in this study. All participants underwent screening for GDM using a 75 g, 2-h oral glucose tolerance test (OGTT) during their routine prenatal clinical visit at 24–28 weeks of gestation. Participants were diagnosed with GDM if their fasting blood glucose exceeded 5.1 mmol/L, or 1-h OGTT value >10.0 mmol/L, or 2-h OGTT value >8.5 mmol/L (22). Among the 50 mothers, 26 were healthy and 24 had GDM. According to the OGTT results, the 24 GDM mothers were classified into three phenotypes: isolated fasting hyperglycemia,isolated postprandial hyperglycemia, and combined fasting and postprandial hyperglycemia (4:16:4). The inclusion criteria were (1) Pregnant women aged 20–44 years (≥20 years and at 24–28 weeks of gestation). (2) Normal pregnancy, with no history of abnormal pregnancies. (3) No major underlying diseases such as tumors. Exclusion criteria were (1) Severe bleeding during pregnancy. (2) Pregnancy complicated with medical diseases. (3) Exposure to harmful substances during pregnancy. This study was approved by the Ethics Committee of the National Institute for Nutrition and Health, Chinese Center for Disease Control and Prevention. All participants signed a written informed consent form. The study has been registered in the Chinese Clinical Trial Registry with the registration number: ChiCTR2400084916.

### 2.2 Data investigation and breast Milk sample collection

#### 2.2.1 Collection of basic information

Basic information was obtained from medical records, including sociodemographic characteristics (age, urban/rural residence), maternal basic information (height, pre-pregnancy weight, weight at delivery, gestational weight gain, delivery mode, parity, gravidity, gestational days), and maternal clinical data (mid-trimester blood glucose, third-trimester blood glucose). For infants, only general sociodemographic characteristics (gender, birth weight, length) were recorded from the medical records.

#### 2.2.2 Collection of breast Milk samples

Breast milk samples were collected at different postpartum stages: 0–7 days, 8–14 days, 1 month, and 3 months postpartum. The collection of breast milk samples from all participants was standardized: samples were obtained between 9:00 and 11:00 a.m., with an interval of at least 2 h after the last breastfeeding, by fully emptying one breast using an electric breast pump. Both lactating mothers and research personnel performed hand and skin disinfection prior to sample collection. Immediately after collection, samples were stored at −18 °C, transported in a validated cold chain box within 24 h, and archived at −80 °C until laboratory analysis.

### 2.3 Quantitative analysis of human milk oligosaccharides

#### 2.3.1 Sample preparation

Frozen breast milk samples were thawed and vortexed, followed by centrifugation at 8,000 rpm at 4 °C. The upper lipid layer was discarded, and the defatted milk was diluted with water. For analysis, an appropriate volume of ethanol was added to the sample solution to remove proteins. The mixture was centrifuged again at 8,000 rpm at 4 °C, and the supernatant was diluted with 50% acetonitrile aqueous solution. The overall dilution ratio of breast milk samples in all solvents was 1:60.

#### 2.3.2 Standard preparation

Thirty-two human milk oligosaccharide reference standards, including 2’-FL, 3-FL, 3’-SL, 6’-SL, LNT, LNnT, LNT-2, DF-L, LNDFH-I, LNFP-I, LNFP-II, LNFP-III, PLNnH, LSTa and LSTc from dsm-firmenich (Heerlen, The Netherlands), as well as DSLNT, 3-*α*-3-FL, LacNAC, LSTb, LSTd, PLNH, LNH, LNDFH-II, LNnDFH-I, LNnDFH, LNnDFH-II, 6’-SLN, 3’-SLN, MFLNH-I, MFLNH-III, DFLNHa and LNnO from Beijing Zhongke Heshengtang Biotechnology Co., Ltd. (Beijing, China), were mixed into a combined standard solution of the desired concentration using 50% acetonitrile, and then diluted 10-fold, 100-fold, and 1,000-fold with 50% acetonitrile, respectively.

#### 2.3.3 Quality control validation

Prior to sample analysis, 1–2 samples were randomly selected and tested to ensure the method was suitable for the batch. A quality control (QC) sample was inserted every 20 samples, with at least three replicates for each QC. The relative standard deviation (RSD) of the three replicates was calculated, with a threshold of ≤15%, to monitor and verify the stability of the instrument and method.

#### 2.3.4 LC–MS analysis

Chromatographic separation was performed on an ACQUITY BEH amide column (130 Å, 1.7 μm, 2.1 × 150 mm) at 50 °C, with a flow rate of 0.3 mL/min and an injection volume of 2 μL. The mobile phase consisted of acetonitrile (A) and 10 mM ammonium formate aqueous solution (B). A linear gradient elution was applied from 0 to 20 min, with the proportion of A decreasing from 90 to 50%, followed by isocratic elution at A/B = 30/70 (v/v) from 20 to 23 min. Mass spectrometric analysis was performed using an Agilent 6,475 triple quadrupole LC/MS system with electrospray ionization (ESI) in negative ion mode. Qualitative and quantitative detection of oligosaccharides was achieved using multiple reaction monitoring (MRM). Key parameters were as follows: capillary voltage 3.0 kV, ion source temperature 350 °C, and collision energy optimized in the range of 2–67 eV according to the characteristics of target analytes. This quantitative method has undergone methodological validation and inter-laboratory comparison to ensure the accuracy of the quantitative results, thereby guaranteeing the reliability and rigor of the detection data (Additional details are provided in Supplementary Table S1).

### 2.4 Statistical analysis

The distribution of continuous variables was evaluated using histograms, skewness and kurtosis measurements, and the Shapiro–Wilk test. Sociodemographic characteristics, anthropometric data of mothers and infants, and HMO concentrations were described as mean ± standard deviation (normally distributed data), median and interquartile range (skewed distributed data), or composition ratio (categorical data). To study the differences between GDM and healthy mothers, two independent sample *t*-tests were used for comparison when the data were normally distributed; Mann–Whitney *U* test (nonparametric test) was used for skewed distributed data; and chi-square test was used for categorical data. Finally, generalized estimating equations (GEE) were applied to investigate the changes and differences in HMOs between healthy mothers and GDM mothers across different lactation stages. A *p*-value <0.05 was considered statistically significant.

## 3 Results

### 3.1 Baseline characteristics of mothers and infants

Basic information of a total of 50 mother-infant pairs was collected, including 26 healthy mothers and 24 GDM mothers. For GDM mothers, the blood glucose concentration measured during the Three times of OGTT (fasting, 1-h, and 2-h) were 4.87 ± 0.44 mmol/L, 9.63 ± 1.55 mmol/L, and 8.31 ± 2.01 mmol/L. The baseline characteristics of the 50 mother-infant pairs are presented in Table 1. Significant differences were observed between healthy mothers and GDM mothers in terms of age, pre-pregnancy BMI, gestational weight gain (GWG), and gestational days (*P*_all_ < 0.05). Specifically, the mean age of healthy mothers was 28.38 ± 2.56 years, compared with 29.95 ± 2.80 years in GDM mothers; the pre-pregnancy BMI of healthy mothers was 21.59 ± 2.38, while that of GDM mothers was 24.40 ± 3.35; the mean gestational days of healthy mothers were 276.46 ± 5.17 days, versus 271.70 ± 6.81 days in GDM mothers. Regarding infant characteristics, there were no significant differences in baseline information between infants born to the two groups of mothers.

**Table 1:** Baseline characteristics of mothers and infants.

Indicator	Healthy mothers (*n* = 26)	GDM mothers (*n* = 24)	*p*-value
Maternal characteristics			
Age, years	28.38 ± 2.56	29.95 ± 2.80	0.044*
Residence, *n* (%)	Rural: 7 (26.92%) Urban: 19 (73.08%)	Rural: 7 (29.17%) Urban: 17 (70.83%)	0.860
Pre-pregnancy weight (kg)	57.42 ± 7.79	63.77 ± 9.66	0.013*
Weight at Delivery (kg)	72.15 ± 8.82	75.87 ± 9.99	0.169
Pre-pregnancy BMI (kg/m^2^)	21.59 ± 2.38	24.40 ± 3.35	0.001*
Gestational weight gain (kg)	14.73 ± 4.10	12.09 ± 3.99	0.026*
Parity	1.00 (1.00–2.00)	2.00 (1.00–2.00)	0.058
Gravidity	1.00 (1.00–2.00)	2.00 (1.00–3.00)	0.296
Mode of delivery, *n* (%)	Vaginal: 10 (38.46%) Cesarean section: 16 (61.54%)	Vaginal: 7 (29.17%) Cesarean section: 17 (70.83%)	0.488
Gestational days	276.46 ± 5.17	271.70 ± 6.81	0.008*
Fasting blood glucose (mmol/L)	4.34 ± 0.28	4.87 ± 0.44	<0.001*
1-h blood glucose (mmol/L)	7.41 ± 1.31	9.63 ± 1.55	<0.001*
2-h blood glucose (mmol/L)	6.58 ± 1.09	8.31 ± 2.01	0.001*
Third trimester blood glucose (mmol/L)	4.37 ± 0.49	4.80 ± 0.92	0.06
Second trimester blood glucose (mmol/L)	4.42 ± 0.40	5.13 ± 0.88	0.002*
heterogeneous GDM phenotypes	–	IFH:4 (16.67%) IPH:16 (66.67%) CFPH:4 (16.66%)	–
Infant characteristics			
Sex, *n* (%)	Male: 15; Female: 11	Male: 13; Female: 11	0.534
Birth weight (g)	3348.92 ± 306.94	3523.33 ± 421.39	0.340
Birth length (cm)	50.00 (50.00–50.00)	50.00 (49.00–50.00)	0.446
Head circumference (cm)	50.00 (48.00–50.00)	50.00 (50.00–50.00)	0.707

*Indicates a statistically significant difference (*p* < 0.05). IFH, Isolated fasting hyperglycemia; IPH, Isolated postprandial hyperglycemi; CFPH: Combined fasting and postprandial hyperglycemia.

### 3.2 Genotype of lactating mothers

Secretor status and Lewis blood type were determined according to the genotyping method for lactating women described by Ren et al. (2). The presence of the product ion m/z 325 from 2’-FL, lactodifucotetraose (LDFT), LNFP-I, and lacto-N-neo-difucohexaose I (LNnDFH-I) was used as an indicator of secretor status, while LNFP-II with the fragment ion m/z 348 was used as an indicator of Lewis blood-group phenotype. In this study, among the 50 mothers, 45 had the (Se + Le+) genotype, 4 had the (Se-Le+) genotype, and 1 had the (Se-Le-) genotype. In the GDM mother group, 20 (83.33%) mothers carried the (Se + Le+) genotype, 3 (12.50%) had the (Se-Le+) genotype, and 1 (4.17%) had the (Se-Le-) genotype. In the healthy mother group, 25 (96.15%) mothers were of the (Se + Le+) genotype, and 1 (3.85%) was of the (Se-Le+) genotype. The impact of maternal genotype on HMOs cannot be ignored. However, due to the extremely small sample size of mothers with the Se-Le + and Se-Le- genotypes in this study, the data lack representativeness. Therefore, subsequent statistical analyses were conducted using data from the dominant (Se + Le+) genotype only. Details are shown in Table 2.

**Table 2:** Distribution of maternal genotypes among study participants in danyang.

Genotype	Healthy mothers (*n* = 26)	GDM mothers (*n* = 24)
Se + Le+	25 (96.15%)	20 (83.33%)
Se-Le+	1 (3.85%)	3 (12.50%)
Se-Le−	0 (0%)	1 (4.17%)

### 3.3 The concentrations and differences in total human milk oligosaccharide between healthy and GDM mothers with the se + Le + genotype

**Figure 1:**
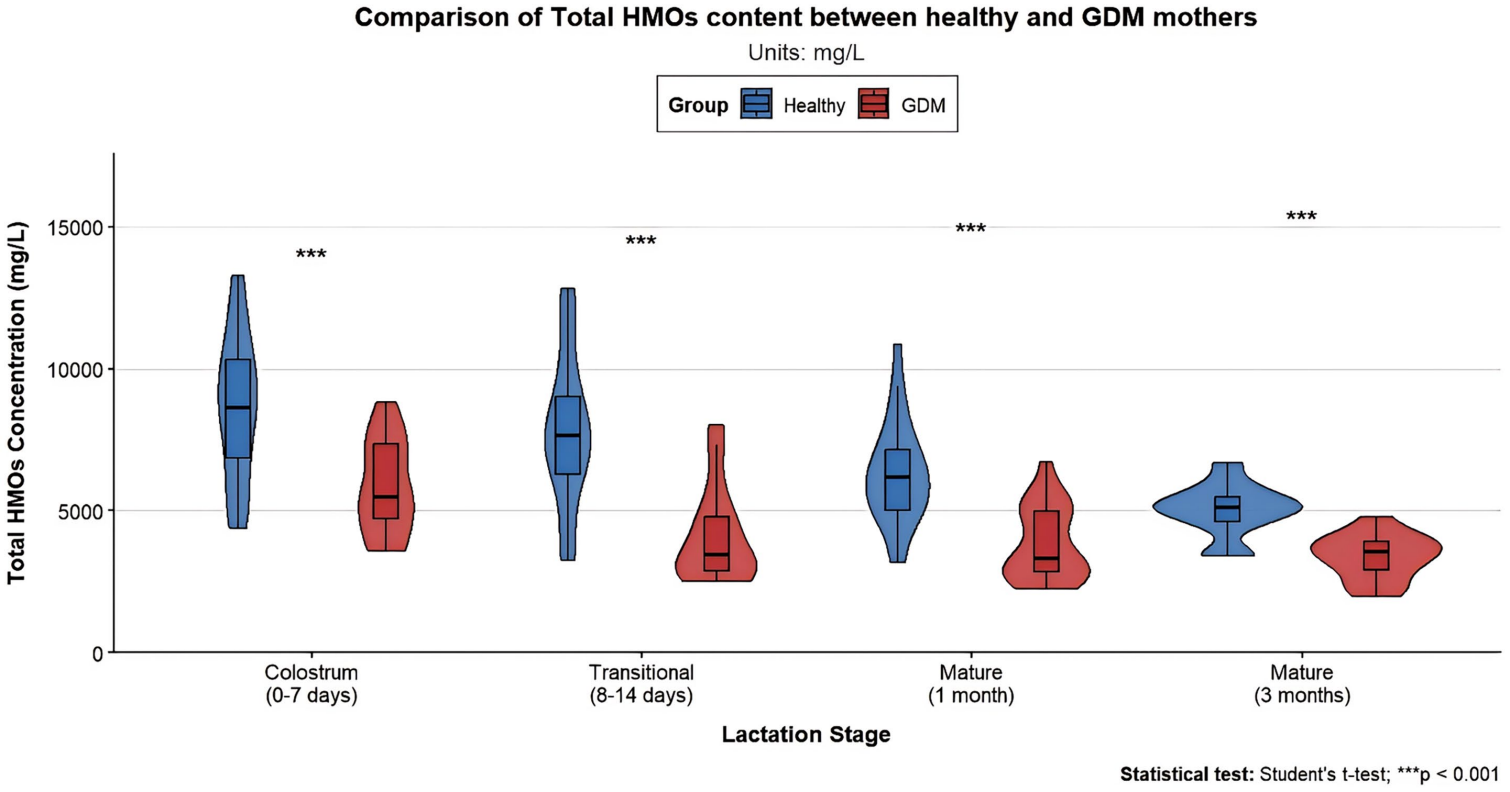
Concentration of total HMOs in breast milk between healthy and GDM mothers with Se + Le + genotype.

Total HMOs concentration is expressed as the sum of 32 HMOs. In healthy mothers, the total HMOs concentration at 0–7 days, 8–14 days, 1 month, and 3 months were 8621.43 ± 2548.47 mg/L, 7849.05 ± 2724.96 mg/L, 6313.56 ± 1810.13 mg/L, and 5014.25 ± 936.89 mg/L. Respectively, in GDM mothers, the total HMOs concentration for the corresponding time periods were 5821.22 ± 1648.50 mg/L, 4104.07 ± 1679.96 mg/L, 3829.55 ± 1297.94 mg/L, and 3366.82 ± 790.93 mg/L. The total HMOs concentration showed a decreasing trend in both groups (Figure 1), and a significant difference in total HMOs concentration was observed between healthy and GDM mothers at all time periods (*P*_all_ < 0.001).

### 3.4 Composition and differences of individual human milk oligosaccharides between healthy and GDM mothers with the se + Le + genotype

**Figure 2:**
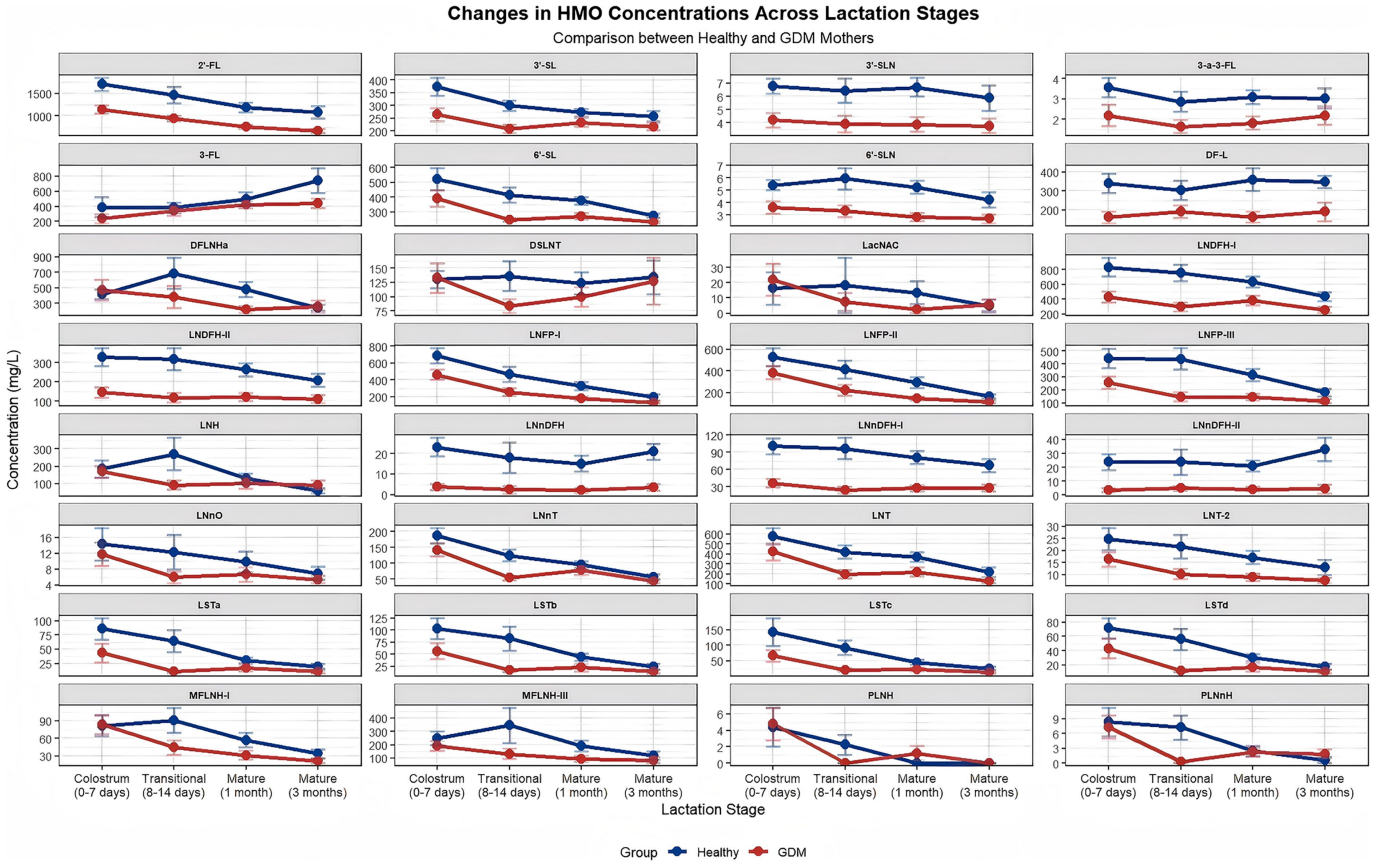
Concentrations and changing trends of individual HMOs in breast milk from healthy and GDM mothers with Se + Le + genotype across lactation stages.

**Figure 3:**
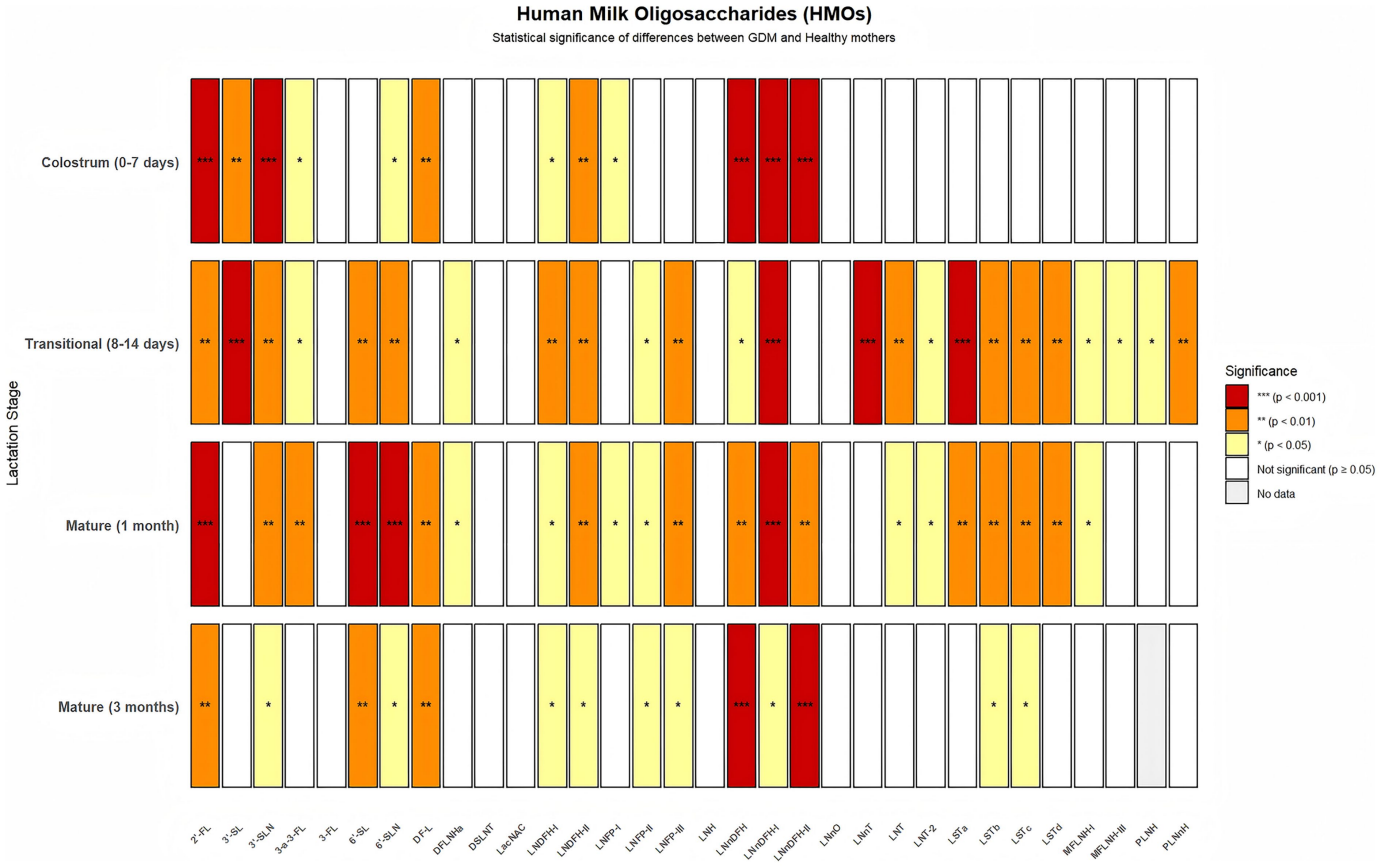
Heatmap of differences in 32 HMOs between healthy and GDM mothers with Se + Le + genotype across lactation stages.

The concentrations of 32 types of HMOs in healthy and GDM mothers at different lactation stages are shown in Figure 2 and Supplementary Table 2. The concentrations of 32 HMOs were measured and it was found that there were significant differences in the concentrations of 2’-FL, LNDFH-I, LNDFH-II, LNnDFH-I, 3’-SLN and 6’-Sialyl-N-acetyllactosamine (6’-SLN) between healthy mothers and GDM mothers at 0–7 days, 8–14 days, 1 month and 3 months (*p* < 0.05). Differences in other HMOs between healthy and GDM mothers were also observed at specific lactation stages. The specific differences are shown in Figure 3. For all HMOs that showed a significant difference, the concentrations were higher in healthy mothers than in GDM mothers. In contrast, the concentration of several HMOs, such as 3-FL, DSLNT, LacNAC, Lacto-N-hexaose (LNH), and Lacto-N-neooctaose (LNnO), did not differ significantly (*p* < 0.05) between these two groups.

### 3.5 Results from the GEE models for HMOs concentration

Generalized Estimating Equations (GEE) were applied to examine the changes and differences in HMOs concentration between healthy and GDM mothers across different lactation stages. The model was specified with an identity link function and a first-order autoregressive (AR) working correlation structure. After removing incomplete cases via listwise deletion and adjusting for covariates (maternal age, BMI, gestational age at delivery, mid-pregnancy blood glucose concentration, and gestational weight gain), multicollinearity analysis was performed for all covariates (Supplementary Table 3). All variance inflation factor (VIF) values were below 2, confirming no significant multicollinearity among variables. The results are presented as follows.

1. Significant Main Effect of GDM Status: After adjusting for time and other covariates, FDR correction for multiple testing was applied. A significant main effect of GDM status was observed on total HMOs concentration (*p =* 0.026). Specifically, the total HMOs concentration were lower in GDM mothers compared to healthy mothers. For different types of HMOs, GDM status showed a significant negative effect on 7 different HMOs: 2’-FL, LNDFH-I, Di-Fucosyl-Lactose (DF-L), LNDFH-II, LNnDFH-I, 3’-SLN, and 6’-SLN (*P*_all_ < 0.05). The detailed results are presented in Table 3.
2. Significant Main Effect of Time: After controlling for the influence of grouping variables (GDM) and other covariates, a significant main effect of time was observed. Compared to the colostrum stage, the total HMOs concentration showed a significant decrease at 1 month and 3 months postpartum (*p* < 0.05). The effect of time varied across different HMOs, as evidenced by a significant negative effect on certain HMOs: the concentration of 2’-FL, LNnT, and LNFP-I at 8–14 days, 1 month, and 3 months were significantly lower than that in the colostrum stage; the concentrations of LNFP-II, LNT-2, Sialyllacto-N-tetraose b (LSTb), Sialyllacto-N-tetraose a (LSTa), Sialyllacto-N-tetraose d (LSTd), Sialyllacto-N-tetraose c (LSTc), Para-Lacto-N-hexaose (PLNH), and Para-Lacto-N-neohexaose (PLNnH) at 1 month and 3 months was significantly lower; and the concentration of 6’-SL, LNT, LNDFH-I, LNFP-III, LNH, 6’-SLN, LNDFH-II, LNnDFH-I, and Monofucosyllacto-N-hexaose III (MFLNH-III) at 3 months was significantly lower than that in the colostrum stage.
3. Interaction between time factor and GDM: The concentration of DSLNT, LacNAC, and Monofucosyllacto-N-hexaose I (MFLNH-I) in mothers of the GDM group showed a greater decline at 8–14 days compared to healthy mothers Figure 2.
4. Influence of Covariates: Age, BMI, gestational age at delivery, mid-pregnancy blood glucose, and gestational weight gain were identified as significant factors correlated with oligosaccharide concentrations (*p* < 0.05). Age presented positive correlations with the concentration of two oligosaccharides 3-*α*-3-FL and 3′-SLN and inverse correlations with DSLNT, LacNAC, and LNnDFH. BMI showed positive associations with LNDFH-I, 3-α-3-FL, and 3′-SLN, and negative associations with DSLNT and LacNAC. Gestational age at delivery was negatively associated with DSLNT and MFLNH-I, and positively associated with 3′-SL. Mid-pregnancy blood glucose was negatively associated with eleven types of HMOs (LNFP-II, 3-α-3-FL, 3′-SLN, LSTa, LSTb, LSTd, LNFP-III, PLNnH, LNH, MFLNH-I, and LNnO), whose concentration decreased with rising glucose. Gestational weight gain was positively correlated with 3-FL and inversely correlated with 2′-FL.

**Table 3:** Effect of gestational diabetes mellitus on human milk oligosaccharides (results from the generalized estimating equations model).

Variable	Estimate	Standard error	95% CI	*Z*-value	*p*-value	Adjusted *p*-value	QIC
Neutral HMOs							
2’-FL	−505.89	185.68	−869.83 ~ −141.96	−2.72	0.006^*^	0.028*	132.05
3-FL	−24.40	193.87	−404.39 ~ 355.59	−0.13	0.899	0.980	125.92
LNT	−23.33	137.77	−293.36 ~ 246.70	−0.17	0.865	0.980	121.51
LNnT	−39.76	37.12	−112.52 ~ 32.99	−1.07	0.284	0.493	126.10
LNFP-I	−205.31	129.44	−441.38 ~ 30.75	−1.70	0.088	0.223	125.67
LNFP-II	−160.23	115.82	−387.24 ~ 66.77	−1.38	0.166	0.351	124.60
LNDFH-I	−404.93	162.90	−724.22 ~ −85.64	−2.49	0.012^*^	0.049*	124.29
DSLNT	32.17	39.19	−44.64 ~ 108.99	0.82	0.411	0.589	128.68
DF-L	−171.60	59.75	−288.73 ~ −54.48	−2.87	0.004^*^	0.026*	128.31
LNFP-III	−204.23	93.65	−387.79 ~ −20.66	−2.18	0.029^*^	0.095	126.12
LNDFH-II	−189.64	53.24	−294.00 ~ −85.27	−3.56	0.0004^*^	0.004*	126.83
LNnDFH	−12.73	5.46	−23.44 ~ −2.02	−2.33	0.019^*^	0.069	123.58
LNnDFH-I	−60.61	17.75	−95.42 ~ −25.80	−3.41	0.0006^*^	0.004*	129.59
Acidic HMOs							
3’-SLN	−3.50	0.99	−5.45 ~ −1.54	−3.51	0.0005^*^	0.004*	126.92
6’-SLN	−2.65	0.66	−3.96 ~ −1.35	−3.99	<0.001^*^	0.003*	126.37
3’-SL	−81.00	56.33	−191.42 ~ 29.42	−1.44	0.150	0.351	130.78
6’-SL	−19.79	95.73	−207.43 ~ 167.83	−0.21	0.836	0.980	126.21
LSTa	−28.87	25.30	−78.47 ~ 20.72	−1.14	0.253	0.463	131.91
LSTd	−17.66	20.06	−56.98 ~ 21.65	−0.88	0.378	0.589	132.84
LSTb	−33.36	27.59	−87.44 ~ 20.71	−1.21	0.226	0.438	130.79
LSTc	−38.95	47.06	−131.18 ~ 53.28	−0.83	0.407	0.589	128.90
Total HMOs	−2225.1	791.71	−3776.83 ~ −673.36	−2.81	0.0049^*^	0.026*	126.63

Healthy mothers were used as the reference group. *Indicates statistical significance (*p* < 0.05). Adjusted *p*-value were calculated using the false discovery rate (FDR) method for multiple testing correction. Among the 32 HMOs analyzed, only those with statistical significance and biological importance are displayed in the table.

## 4 Discussion

This study focused on the variations and differences in HMOs between GDM mothers and healthy mothers, extending the scope from primary HMOs to 32 individual HMOs. GEE analysis revealed that the total HMO concentration, as well as the concentrations of seven specific HMOs, including 2’-FL, LNDFH-I, and 3’-SLN, differed significantly between GDM and healthy mothers across different lactation stages (*P*_all_ < 0.05), with lower concentrations observed in the GDM group. Meanwhile, we further conducted analyses using mixed-effects models, and the results were generally consistent with those of the GEE model. The two analytical methods showed generally consistent results, further verifying the robustness of statistical models and the reliability of our findings. Detailed results of the mixed-effects model are presented in Supplementary Table 3. As shown in Table 3, GDM was associated with decreased concentrations of most neutral HMOs, while among acidic HMOs, only 3’-SLN and 6’-SLN were significantly associated with GDM, with their concentrations being correspondingly reduced. These results are similar to previous studies by Ye et al. (17), Wang et al. (18) and Zhang et al. (21), which also reported lower concentrations of certain HMOs in GDM mothers. It is also consistent with the research of Li et al. (23), which found significantly lower total and specific HMO concentrations in colostrum from GDM mothers compared with healthy controls. However, our results differ from some studies, such as Smilowitz et al. (16), who reported no significant differences in HMO concentrations between GDM and healthy mothers in transitional milk collected at 2 weeks postpartum, and Dou et al. (20), who observed significantly higher concentrations of certain HMOs in GDM mothers. These discrepancies may be related to geographical factors and variations in the proportional distribution of heterogeneous GDM phenotypes across study cohorts.

In addition to exploring the associations between GDM and HMO concentrations, this study observed that most HMOs exhibited a declining trend over the course of lactation. For example, the concentrations of 2’-FL, LNnT, and LNFP-I in both transitional and mature milk were significantly different from those in colostrum. The concentrations of 17 specific HMOs, including 6’-SL and LNT, also showed significant differences between mature milk and colostrum, with concentrations decreasing over time. These findings are consistent with some previous studies (24). However, an increasing trend in 3-FL over the lactation period was not observed in this study, which may be attributed to the relatively small sample size, regional factors, maternal dietary habits, or other variations.

In our GEE analysis of the effects of GDM on HMOs, we also exploratively identified maternal age, BMI, gestational age at delivery, mid-pregnancy blood glucose, and gestational weight gain as factors associated with HMO concentrations. An international cross-sectional cohort study involving 410 mothers reported associations between maternal age, BMI, and several HMOs; for instance, age was negatively correlated with LNnT, LSTc, and DSLNH concentrations but positively correlated with FLNH, while maternal BMI was negatively correlated with DSLNT concentration (25). Our study identified significant inverse associations between mid-pregnancy blood glucose and 11 oligosaccharides, including LNFP-II, 3-a-3-FL, and 3’-SLN. Therefore, we tentatively hypothesize that maternal metabolic characteristics may influence the variability of HMO profiles. All the above maternal indicators were adjusted for as confounding factors, allowing us to independently evaluate the association between GDM and HMO profiles. Current evidence regarding the influences of maternal metabolic conditions on HMOs is still accumulating. These observations may help inform the adjustment and control of potential confounding factors in future investigations into the associations between GDM and HMO profiles.

Our study found that GDM is correlated with lower levels of specific HMOs, many of which have been demonstrated to possess immunomodulatory, intestinal function-regulating, and prebiotic properties (26–28). Given the known biological functions of these HMOs, altered concentrations in GDM mothers may have potential implications for maternal and infant health, although clinical outcomes were not assessed in the present study. Therefore, more research is needed to explore the potential pathways connecting GDM with altered HMO profiles, as well as the subsequent relevance to maternal and infant health, in order to better prevent and mitigate the negative consequences of GDM for mothers and infants.

Based on a review of the literature, the impact of GDM on HMO concentrations may be attributed to the following three potential mechanisms. Firstly, changes in HMO concentrations in mothers with GDM may result from alterations in the activity of key enzymes, particularly glycosyl transferases and glycosidases. Insulin resistance and hormonal dysregulation associated with GDM could modulate the activity of these enzymes, thereby inhibiting HMO synthesis and leading to a decrease in HMO concentrations (29), although this mechanism requires further investigation. Secondly, elevated blood glucose concentration in GDM patients may significantly increase Toll-like receptor expression in monocytes, activating inflammatory transcription factors and enhancing pro-inflammatory cytokine secretion, resulting in a state of chronic low-grade inflammation (30), which is a key pathological feature of GDM (31). Under such chronic inflammatory conditions, elevated inflammatory cytokines can impair insulin signaling, reduce insulin sensitivity, and exacerbate insulin resistance. This dysregulation might in turn alter the activity of glycosyltransferases and glycosidases, which could thereby affect HMO concentrations (29). Thirdly, GDM may influence maternal gut microbiota, leading to dysbiosis, characterized by an increased abundance of Firmicutes, Ruminococcus, and *Escherichia coli*, alongside a reduction in beneficial bacteria such as Bacteroides, Bifidobacterium, and Lactobacillus (32–34). Gut microbiota dysbiosis may disrupt the intestinal barrier, induce systemic inflammation, and promote bacterial translocation along the “gut-mammary axis.” These cascading changes could hypothetically interfere with HMO metabolic turnover, compromise HMO synthetic capacity, and ultimately tend to reduce HMO abundance. This speculative pathway is partially implied by previously reported correlations between specific gut microbes and HMO profiles (35, 36). Given that GDM is a systemic metabolic disorder with complex effects on maternal physiology, most current research has focused on the aforementioned aspects. Further research is still needed to explore its influence on other metabolic pathways, such as energy metabolism, to gain deeper insights into how GDM affects HMO concentrations.

This study has several limitations. Firstly, the study population consisted of only 50 mothers from Danyang City, Jiangsu Province, across different lactation stages, resulting in a relatively small sample size. Secondly, the majority of participants were secretor-positive mothers, with mothers of other genotypes being underrepresented. Since genotype significantly influences HMO concentrations, the present analysis was restricted to Se⁺Le⁺ mothers to ensure accuracy, thereby precluding any comparisons of HMO profiles across different genotypes. This decision further aggravates the limitation of the small sample size and reduces the generalizability of our findings to lactating women with other genotypes. Future studies with larger sample sizes are warranted to explore the alterations and differences in HMOs among GDM mothers with distinct genotypes. Thirdly, this study classified GDM into three heterogeneous phenotypes based on OGTT results. However, in the current cohort, most women with GDM exhibited isolated postprandial hyperglycemia, while the cases of the other two subtypes were relatively scarce. Therefore, stratified statistical analysis for each GDM phenotype could not be reliably conducted. In the future, we will recruit a larger study cohort with balanced distribution of GDM phenotypes to further compare HMO profiles across different GDM subtypes. Finally, there are certain limitations in the statistical methods: first, although multiple covariates have been adjusted for in this study, the impact of unmeasured confounders on HMOs cannot be completely ruled out; second, the introduction of covariates and interaction terms increases the complexity of the statistical model, which may pose a slight risk of overfitting; third, as an observational study, this research can only reveal associations and cannot fully eliminate the limitations inherent in causal inference. Despite these limitations, this study comprehensively compared the changes and differences in 32 HMOs between mothers with GDM and healthy controls across four lactation time periods (0–7 days, 8–14 days, 1 month, and 3 months), and ensured the stability of the model through multi-model comparison to improve the reliability of the conclusions. The findings provide a valuable reference for future larger and more extensive cohort studies on the relationship between GDM and HMOs.

## Data Availability

The datasets presented in this study can be found in online repositories. The names of the repository/repositories and accession number(s) can be found in the article/Supplementary material.
